# Neighborhood Physical Environment and Cognition in Later Life: A Cross-Sectional Study in Hong Kong

**DOI:** 10.1093/geroni/igab046.1811

**Published:** 2021-12-17

**Authors:** On Fung Chan, Yuqi Liu, Yingqi Guo, Shiyu Lu, Hiu Kwan Chui, Lai Har Chiu, Chris Webster, Terry Y S Lum

**Affiliations:** The University of Hong Kong, Hong Kong, Hong Kong

## Abstract

This study examined the association between neighbourhood physical environment and cognition among community-dwelling older people and identifies whether this association varies among different older age groups. Data came from a cross-sectional survey data with 2,081 older people living in 12 public housing estates in Hong Kong. We merged individual data with neighbourhood physical environment data from community audit and GIS. Multivariable linear regression model was used. Young-old who resided in neighbourhoods with a higher land use mix and more public transport terminals, were associated with better cognition. Only the number of community centres was positively associated with the cognition for old-old. A curvilinear association was found between cognition and the number of active leisure facilities in the overall sample and young-old. Our findings could inform urban planners and policymakers on planning community facilities and physical environments based on the needs of older people in different age groups.

